# Parent’s Perspective on Continuity of Care in the Maternity Care and Child Health Services Continuum: A Qualitative Systematic Review

**DOI:** 10.5334/ijic.8645

**Published:** 2025-01-24

**Authors:** Anne C. M. Hermans, Silke Boertien, Lauri M. M. van den Berg, Ank de Jonge, Danielle E. M. C. Janssen, Arie Franx, Jacoba van der Kooy, Marlou L. A. de Kroon

**Affiliations:** 1 Department of Obstetrics and Gynaecology, Erasmus MC—Sophia Children’s Hospital, Rotterdam, The Netherlands; 2 Department of Midwifery Science, AVAG/Amsterdam Public Health, Amsterdam University Medical Centre, Vrije Universiteit Amsterdam, Amsterdam, The Netherlands; 3 Department of General Practice and Elderly Care Medicine, University Medical Centre Groningen, University of Groningen, Groningen, The Netherlands; 4 Department of Health Sciences, University of Groningen and University Medical Centre Groningen, Groningen, The Netherlands; 5 Department of Public Health and Primary Care, Environment and Health, Youth Health Care, University of Leuven, KU Leuven, Leuven, Belgium

**Keywords:** systematic review, postpartum care, postnatal care, maternity care, child health care services, continuity of care, parent perspective

## Abstract

**Introduction:**

While the World Health Organization (WHO) advocates organizing maternity care and preventive child healthcare (PCHC) as people-centred, integrated healthcare services, globally these services are often established separately, causing discontinuity of care. Our aim is to synthesize the evidence concerning what impacts parents’ experience of continuity of care, and how to promote it.

**Methods:**

Qualitative systematic review. Embase, Medline, Web of Science, Cochrane, CINAHL and Google Scholar were searched for studies on parents’ perspectives on integrated care. Helpful practices and issues regarding continuity of care were identified.

**Results:**

We found that parents valued easily accessible, tailored, family-centred care that is a display of interprofessional collaboration and is geared towards supporting and empowering parents.

**Discussion:**

Study strengths are its qualitative nature, allowing for in-depth patient views and experiences, and the multidisciplinary research team, which ensured a multidimensional view of the issue.

**Conclusion:**

Ideally, (a) parents enter the postnatal period well-prepared, and well-informed about self-care, PCHC and possible postnatal carepathways, (b) number of caretransfers is limited, (c) by overlapping maternity care and PCHC, parents are provided with an opportunity to maintain meaningful relationships with their care providers, and (d) information is consistent, family-centred, and tailored.

## Introduction

While the World Health Organization (WHO) advocates organizing maternity care and preventive child healthcare (PCHC) as people-centred, integrated healthcare services, globally these services are often established separately [1,2], leading to discontinuity of care due to fragmented care delivery [1,2,3].

The terms ‘integrated care’ and ‘continuity of care’ are related, however, not the same. Experiencing continuity of care is part of integrated care, but integrated care further entails ‘the ability to effectively coordinate care around people’s needs’ [4].

Despite recognition of the importance of integrated care and interprofessional collaboration, parents are generally less satisfied with postnatal care compared to prenatal care due to a lack of continuity in addressing individual needs, leading to unmet healthcare needs and limited access during the transition to parenthood [5,6,7,8]. The first 1000 days of a child’s life (from ten months before birth up to the child’s second birthday) are crucial for lifelong health and development, and are influenced by maternal sensitivity, proper nutrition, and cognitive engagement. [9,10]. However, the postnatal period is marked by parental challenges with emotional vulnerabilities [11,12,13].

Experiencing continuity of care is important in the postnatal period to provide parents with a sense of security [14]. Hence the interest in strategies to integrate maternity care and Child Healthcare (CHC) [15] which promote interprofessional collaboration and integrate care services to ensure continuity of care and to meet the family needs during the postnatal period [16].

Yet, interprofessional collaboration faces challenges [17] as providers often differ in values, culture, and clinical approaches. Separate financial systems and conflicting regulations further hinder integration [18].

Understanding parents’ perspectives on integrated care is essential to promote continuity. Our aim is to synthesize the evidence concerning what impacts parents’ experience of continuity of care, and how to promote it.

## Methods

### Search Strategy

**Figure 1:**
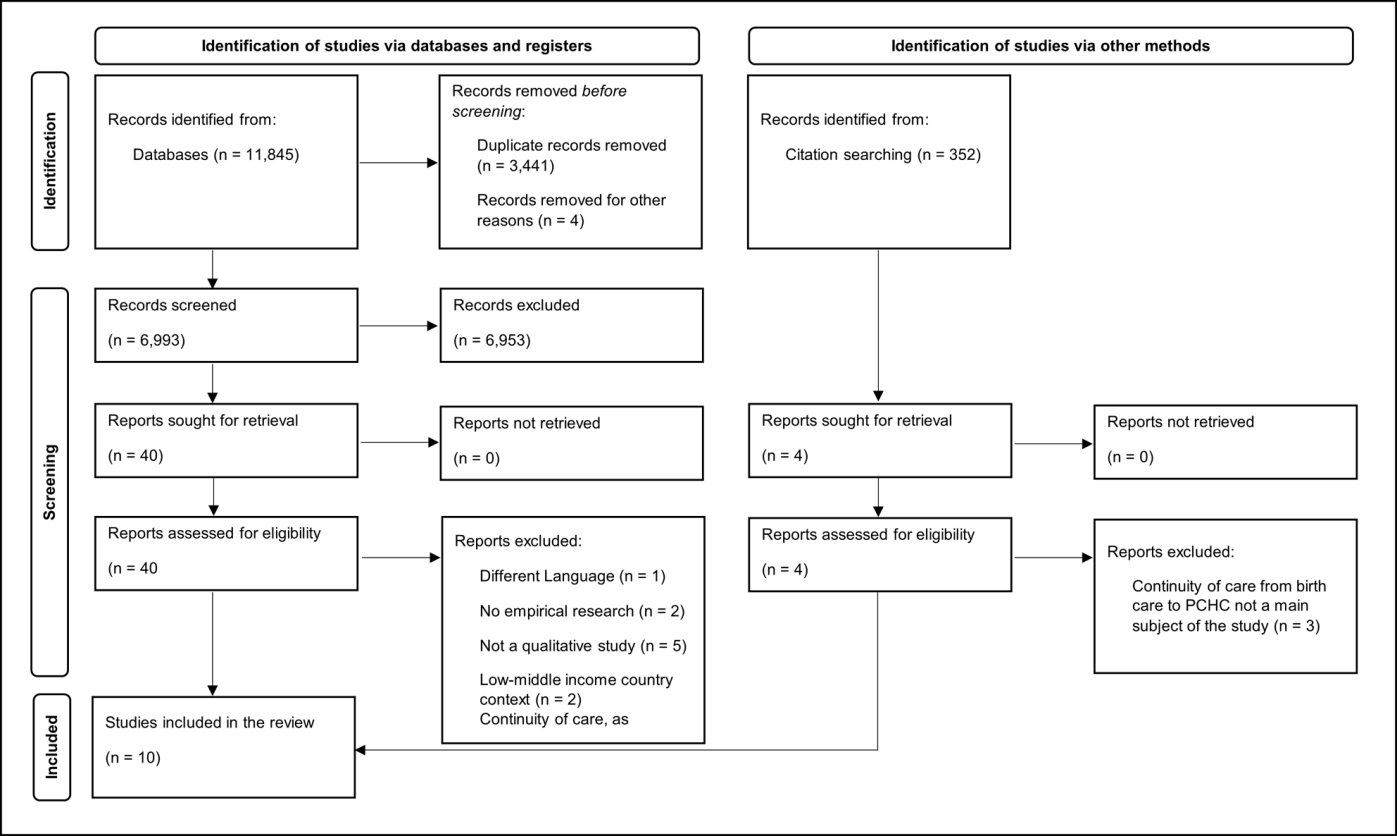
PRISMA flow diagram.

Six electronic databases were searched (February 2021 and May 2022): Embase, Medline, Web of Science, Cochrane Central Register of Controlled Trials, CINAHL and Google Scholar. Included terms were related to: 1) ‘continuity of care’/‘integrated care’, 2) ‘maternity care’, 3) ‘preventive child health services’, 4) ‘postnatal period’ and 5) ‘parent perspective’. Figure 1 demonstrates the study selection process. Appendix A includes the full MEDLINE search strategy. No time filter restriction was applied, as this could lead to unwanted exclusion of relevant studies [19]. The search strategy was developed by SB, MdK developed the search strategy. SMG (biomedical information specialist from Erasmus Medical Centre) conducted the search.

New terms were added to the second search, which were derived from previous included articles. The search was updated in October 2024.

### Inclusion criteria

We included qualitative research papers [20] that met following criteria: the study was 1) an original research paper published in a scientific journal, 2) written in English, 3) conducted in a high-income country with publicly available maternity care and child health services, and 4) mainly about the perspective of parents on integration of care and/or continuity of care (during the transition from maternity services to PCHC). Articles were excluded when they were focussed on perspectives on either maternity care or PCHC.

Maternity care and PCHC are organised in various ways. Therefore, the professional roles and responsibilities may vary. To systematically determine whether the inclusion criteria were met, SB and LvdB agreed upon (broad) definitions of both maternity care and PCHC. Maternity care was defined as all care that is provided before, during and up to 3 months postpartum by obstetricians, midwives, physicians, paediatricians or neonatologists. PCHC care was defined as care organized to offer universal preventive healthcare to children (until at least 1 year of age), often provided through consultations at well-baby clinics or paediatricians.

### Selection process

After the first search SB and LvdB independently screened titles and abstracts of 5840 articles. Then, selected articles were read and assessed independently. Disagreements were resolved via consensus or consultation of MdK. Afterwards, SB searched the reference lists for potential additional papers. Disagreements were resolved through discussion. This process was repeated in May 2022.

### Quality assessment

Study quality was independently assessed and discussed by SB and LvdB using the Standards for Reporting Qualitative Research (SRQR) checklist (supplemental Table 1, Appendix A) which includes 21 items that are considered essential for complete reporting of qualitative research [21]. The study quality was taken into consideration when evaluating the results.

### Data extraction and synthesis

Extracted data included aim(s), methods, relevant findings (Table 1) and issues complicating continuity and helpful practises promoting continuity mentioned in the article. After individual familiarization with the data and extensive discussion, SB and LvdB constructed four themes [22]. SB wrote the narrative synthesis, which was further refined by all authors.

**Table 1:** Summarized aims, methods, and findings of all included studies.


NR.	REFERENCE	AIM	METHODS	PARTICIPANTS AND SETTING	RESULTS RELATING TO CONTINUITY OF CARE

**1**.	Barimani and Hylander (2012)	To investigate strategies to improve continuity of care for expectant and new mothers and to elaborate on a preliminary substantive grounded theory model of “linkage in the chain of care” which was developed by Barimani and Hylander in 2008.	Data collection: structured interviews, participant observation and document analysis Sample: theoretical	Mothers who visited a family health centre with clear collaboration policies (n = 11) Mothers who visited a medical centre without such policies (n = 10) Country: Sweden	Midwives and CHC professionals working in the same building does not guarantee collaboration. Mothers clearly notice when CHC professionals and midwives work as a team and are very positive about teamwork. Mothers appreciate to have both a pre- and postnatal relationship with both midwife and CHC professional. Collaborative home visits were highly appreciated as well as joined policies and breastfeeding support. Continuity of care policies seem to lead to a more positive care experience.

**2**.	Barimani et al. (2015)	To explore ways in which parents experience support from health professionals in the early postpartum period and understand how parenting support is related to management, informational, and relational continuity.	Data collection: Focus group interviews Sample: convenience	Mothers (n = 18) and fathers (n = 16) between 20–46 years of age form urban area with one or more children, whose youngest child is ≤ 1 year old. Country: Sweden	With regard to management continuity parents value: receiving consistent advice, knowing who to ask questions regarding care, getting access to care when needed, feeling confident on various pathways and being involved in discharge planning. With regard to informational continuity parents value: receiving consistent information about self-management for both mother and baby and being was valued. Regarding relational continuity parents value: team or clinic care consistency, trust in a specific person, interest in mother from CHC, inter-pregnancy continuity of carer and later withdrawal of midwifery care and earlier introduction of the CHC were valued.

**3**.	Vikström and Barimani (2016)	To explore (i) ways in which partners experienced support from care systems before, during, and after childbirth in relation to their parenting roles and (ii) their suggested ways to improve postnatal support.	Data collection: focus groups Sample: convenience	Partners of mothers who had given birth in a Swedish hospital, with one or more children, whose youngest child is ≤ 1 year old who read and speak Swedish (n = 17). Country: Sweden	Follow-up appointments with the postnatal care unit provide a sense of security. However, the postnatal care unit only felt accessible to the participants during the first week after the birth. After being discharged parents feel child health and medical care are prioritized most. (Mental) care for the partners is missed. Conflicting advice (specifically about breastfeeding) given by health care providers creates uncertainty. Partners might also benefit from follow up, such as routine mental screening.

**4**.	Woodward, Zadoroznyj, Benoit (2016)	To provide qualitative insights into women’s experiences of the different forms of postnatal care in the community, and identify where improvements could be made to service provision.	Data collection: semi structured interviews Sample: purposive	Mothers whose youngest child is ≤ 1 year old, who have used one of the different postnatal services in Australia (n = 15). Country: Australia	It is import for mothers to know what follow up will look like and where they will be able to find support. When this lacks, mothers experience a gap in care. Structured postnatal follow-up provides parents with information, reassurance and confidence. Continuity of relationships is important for mothers to build trust. Mothers would appreciate women-centred care, also after birth. Antenatal care is felt to be more women-centred than care provided at the CHC clinic. Mothers will seek support elsewhere (pharmacy or GP) when they feel the care at the CHC clinic lacks.

**5**.	Franck, McNulty and Alderdice (2017)	To discover parents’ views, experiences, concerns, and recommendations about the care provided to them and their babies throughout the perinatal and neonatal healthcare journey in a UK context.	Data collection: focus groups Sample: convenience	Mothers (n = 33) and fathers (n = 7) of babies who received care in 1 of the 7 NICUs in Northern Ireland within the past 3 years. Country: Northern Ireland	Mother and child being discharged at different times can lead to problems with follow-up care. Going home after being in the NICU environment can be a scary transition. Parents feel they have become dependent on care personnel to care for their baby. Feeling excluded from decision making or lacking access to information about child care negatively impacts parents and their relationship with their child. Mental health issues can present themselves after post discharge support services have been withdrawn. Participants are distressed by a lack of continuity of carer, incomplete transfers of information and inconsistencies in information and care practices. After leaving the NICU parents could feel discouraged to contact the NICU when problems occurred. CHC professional and communal midwifes sometimes lack specific knowledge about the growth and care of premature babies and lacked confidence in care for them. Parents would like better access to a community midwife or health visitor, when they get home from the NICU.

**6**.	Aquino, Olander and Bryar (2018)	To explore women’s (i) experiences of maternity care as collaboratively provided by midwives and CHC professionals, and (ii) their perspectives of how their maternity care can best be provided by these healthcare professionals together.	Data collection: focus groups Sample: convenience	Mothers ≥ 18 years of age with a child ≤ 18 months old who read and speak English (n = 12). Country: England	Mothers observe there was little to no contact between the midwife and the CHC professional. Disagreements between care providers harms confidence in care professionals. In the ideal maternity care pathway the CHC professional is introduced prenatally and the midwife provides care until first month after birth. Continuity of carer is valued, but it is acknowledged it might not be realistic. Centralized records, joint appointments and classes would be appreciated. Clarity on the roles and tasks of health care providers, specifically the public health nurse would be valued.

**7**.	Olander et al. (2019)	To explore recent mothers’ experiences and views of the continuity of information shared and provided by midwives and CHC professionals during and after pregnancy.	Data collection: semi-structured interviews Sample: convenience	Mothers ≥ 18 years of age who have had a baby within 12 months prior to the interview, read and speak English and have had antenatal and postnatal care (n = 29). Country: England	What information is shared between the midwife and the CHC professional, and how is unclear to mothers, who sometimes doubt any information sharing occurs. Mothers are often positive about information sharing. Sharing sensitive information with the CHC clinic is sometimes feared however, due to their (perceived) role as ‘frontline social service’. Having to repeat information is often disliked, especially about traumatic experiences. However, storytelling can also be a way to get to know a healthcare provider. Flexible, individualized care and teamwork are highly valued by the participants. Joint working, training and development of guidelines are suggested as possible solutions to increase continuity of information.

**8**.	Olander, Aquino and Bryar (2019)	To explore midwives,’ CHC professionals and postnatal women’s experiences and views of co-location of midwifery and health visiting services and collaborative practice.	See previous article (same study).	See previous article (same study).	Not all respondents were convinced of the importance of service co-location. Service integration was deemed more important. Midwives and CHC professionals were viewed as having different roles and functioning separately. Possible benefits to co-location could be reducing how often parents need to repeat information and reducing the amount of conflicting advice. Only having to go to one place for all care could also be beneficial for those in difficult social circumstances as it can be more convenient and cheap. It also creates a place to meet other parents.

**9**.	Forss, Mangrio and Hellström (2022)	To illuminate first-time parents’ experience of a home visit conducted by a midwife and a child health care nurse 1–2 weeks postnatal.	Data collection: semi-structured interviews Sample: convenience	First time mothers (13) and fathers (12) who participated in a new combined home visit program. Country: Sweden	It was important to have the known midwife at the home visit. The combined interprofessional knowledge base of the midwife and the CHC nurse made parents feel secure and ensured the needs of the whole family were met. The great availability of the professionals made parents feel secure. Having the CHC professional visit at home creates the possibility to spot possible safety hazards and was found convenient. Mothers and fathers had different needs and appreciated the possibility to discuss their specific concerns with both professionals.

**10**.	Frederiksen, Schmied, Overgaard (2022)	To explore the role of continuity of care in creating a coherent care journey for vulnerable parents during pregnancy and the postnatal period.	Data collection: observation and informal semi-structured interviews Sample: purposive	Mothers (n = 26) and partners (n = 13) who: expect a child or have recently given birth, reside in the municipality under study, speak Danish, and receive high level services due to one or more vulnerability factors. Country: Denmark	Getting to know professionals over time is valued, facilitates reaching out for support and being honest about challenges and provides a sense of security. Meeting new professionals can be scary due to a fear of judgement and stigmatization. Continuity of carer and information transfer allow for more individualized care. A non-judgemental and supportive approach thus allows parents to feel secure with professionals they do not know. Transfer of information enables parents to experience a degree of coherence in their journey and prevents parents from having to repeat information, which can be demanding. Parents should be ensured which information is transferred as knowing information is transferred can make them feel more secure. However, handover of information (to social services) can also be experienced as risky. Care systems need to be flexible and adaptable to meet the fluctuating care needs of parents and prevent gaps in care. To ensure coherent care it is paramount that parents receive timely, flexible and relevant services that match their current needs for support.


## Results

### Study characteristics

The ten included studies (Table 1) were from the UK (3), Australia (1), Sweden (4), Denmark (1), and Northern Ireland (1), published between 2012 and 2020, with two UK articles from the same study. In total, 128 mothers and 40 partners were interviewed. Five studies only included interviews with mothers [23,24,25,26,27], one study only included the partners [28] and four studies included both [29,30,31,32]. Two studies included interviews with care providers (24,33), which we did not analyze. Three studies focused on specific parent groups: first-time parents (30), pre-term births (32), and vulnerable situations (31). Table 1 summarizes aims, methods, and findings of all studies.

### Study Quality

Supplemental Table 1 (Appendix A) demonstrates the quality appraisal. The study quality was moderately good; only three studies detailed their theoretical approach, and four adequately described researcher-participant relationships. This may be due to qualitative research’s underrepresentation and stricter quality standards in recent years [33].

### Findings

The included studies identified various issues and helpful practices regarding continuity of care, along with strategies to address experienced discontinuity, and enhance a continuous care experience. The issues and helpful practices are summarized in Tables 2 and 3, which are structured chronologically. Parents’ perspectives may vary: For instance, practices like joint home-visits by the midwife and PCHC professional, which some parents found beneficial for continuity, were seen as uncomfortable by others [23,24,25]. The different aims, contexts and research populations have contributed to the heterogeneity of the proposed issues and helpful practises. However, also within the studies varying experiences and different perspectives on what is needed to improve continuity were described, regarding care pathways and relationships with healthcare providers. This variety in experiences and perspectives highlights the complexity of promoting an experience of continuity of care for all parents as there are no ‘one-size fits all’-solutions to meet the specific needs of parents. Furthermore, most issues that could impact the parents’ experience of continuity of care did not have one single potential solution, and some of the practises improving continuity of care were helpful in multiple ways. For example joint home visits by the midwife and the CHC care provider might enhance both consistent communication as well as trust between the mother and both healthcare professionals [25]. However, despite the variety, we also found communalities with regard to what mattered to parents’ sense of continuity of care and thus where an impactful, positive change in care could be made. Around these communalities we have constructed four themes, which are discussed in the following narrative analysis.

**Table 2:** Parent-reported issues complicating continuous care, based on reviewed papers.


DURING PREGNANCY	WITHDRAWING MATERNITY CARE	TRANSFERRING CARE BETWEEN PROVIDERS	BEING AT HOME IN BETWEEN SCHEDULED APPOINTMENTS	THE APPOINTMENTS AT THE CHC CLINIC	THE MATERNITY CARE FOLLOW- UP

- Lack of preparation for the postnatal period and lack information on child-care, self-care, the organization of postnatal care (23, 25, 26, 32) - Lack of desired meeting with CHC professional during pregnancy (23, 29, 30, 31)	- The withdrawal of support from the trusted midwife can feel sudden and parents do not always feel ready (23, 26, 29, 32) - Parents are sometimes not actively involved in de discharge/last maternity care visit and insufficient information is shared to be self-reliant and be able to access care easily (25, 28) - Parents can feel like it is not appropriate anymore to contact the hospital/midwife after the first days (27, 29, 32)	- Transfers of care can disrupt the building of trust (23, 25, 28–32) - Services feel fragmented because communication between professionals lacks, is negative or is not visible (23–25, 30–32) - The transfer of care can introduce variation in the quality of care (23, 32) - The transfer of care can result in feeling constantly assessed by new professionals (31) - Poor coordination of care can lead to duplications of care (such as multiple weightings in one day), gaps and poorly scheduled appointments (23, 25, 32) - A poor transfer of information can result in important information being missed, parents having to repeat information, care being experienced as impersonal and can harm the relationship between professional and parent (23–25, 31) - Different professionals sometimes offer different information and advice (23–25)	- Insecurity about child-care and self-care, for which they need advice and/or reassurance (25, 26, 28) - Parents can experience difficulties with finding reliable information (23, 26, 27) - Parents can find it (emotionally) difficult to reach out for support (30, 31) - Parents are not always aware of whom they can contact to find appropriate professional support when problems arise (25, 26, 28, 31) - Due the withdrawal of maternity care and different focus of CHC services it can be unclear how parents can access care for themselves (26, 28, 29) - There can be long waiting times when trying to access care (28, 31)	- A lack of continuity of carer within the organization (26, 30–32) - It can be harder for parents to trust the CHC professional as they are seen as a ‘front line social service’ (25, 31) - Appointments at CHC services are sometimes experienced as impersonal and/or regimented (23, 26, 32) - Appointments at CHC services are sometimes experienced as only focussed on the child’s well-being, leaving parents feeling unsupported (26, 32)	- Standard follow-up does not always sufficiently meet physical and emotional needs of the parents (28) - Not enough or badly timed follow-up appointments (28) - The follow-up appointment is sometimes performed by someone the mother does not know (29, 30, 31) - A lack of attention for and/or monitoring of the emotional well-being of the partner (25, 32)


**Table 3:** Partent-reported helpful practices promoting continuous care, based on reviewd papers (references 23–32).


DURING PREGNANCY	WHEN WITHDRAWING MATERNITY CARE	WHEN TRANSFERRING CARE BETWEEN PROVIDERS	IN BETWEEN SCHEDULED APPOINTMENTS	DURING APPOINTMENTS AT THE CHC CLINIC	DURING THE MATERNITY CARE FOLLOW-UP	LONG TERM ORGANIZATIONAL REFORM

- Provide sufficient information on the postnatal period, child-care, self-care, possible postnatal care pathways and the role of the CHC professional before birth (23, 25, 28) - Introduce the CHC professional before birth (23, 24, 29–31) - Organize group classes or introducing parents to an existing parent gatherings (23, 26, 29, 32)	- Involve parents in the discharge from the hospital/last midwifery care appointment at home after a home birth and providing parents with elaborate information on accessing care (for all family members) including contact information (25, 26, 28, 32) - Ensure parents have received sufficient self-management information (28, 32) - Create a low threshold to re-establish contact with the maternity care provider (25, 28–31)	- Introduce the CHC professional with the known maternity care provider present (23, 24, 29) - Combine appointments with both maternity carer and the CHC professional (23–25, 29, 30) - Coordinate care well to avoid duplications, gaps and poorly scheduled appointments (23, 25, 32) - Timely transfer important information, with parental consent (23, 25, 29, 30) - Make collaboration and the transfer of information transparent and visible (23, 25, 29) - Visibly show mutual respect for each other’s expertise (24, 30)	- A (jointly organized) telephone service to offer support, reassurance and advice (23, 28, 32) - Offer reliable internet sources/online chat service/forum to ask questions (23, 27, 28) - Make sure parents feel it is normal and not a problem when to need ask for help (23, 28) - Make it easy to timely schedule and reschedule appointments (28) - Introduce a well-woman check or check of maternal physical health at the General Practitioner (23)	- Use CHC appointments to also check up on the (emotional) well-being of the parents (23, 26, 28) - Use an individualized and family-centred approach (23, 26, 31, 32) - Ensure continuity of carer over different appointments and pregnancies (23, 26) - Carefully read shared information (32)	- Let a known maternity carer perform the follow-up appointment (23, 26–28, 30–32) - Make sure to meet all the parents’ needs, including emotional needs (27–31) - Match the date of the follow-up appointment with the parent’s needs and/or offer multiple check up’s (25, 27, 28, 31, 32) - Pay explicit attention to the concerns and emotional wellbeing of partners (27, 30, 32)	- Consider co-location (24, 29) - Digitalize information through centralized records (23, 25) - Work towards consensus on care standards, policies in order to be able to provide consistent advice across providers (23–25) - Organizing joint (learning) activities with maternity care and CHC service, also specifically to improve knowledge on preterm babies (24, 29, 32)


#### Theme 1: Parents’ wish for feeling secure and supported at home

An important identified issue was that parents did not always feel secure and supported in the postnatal period, due to a gap in care between maternity care and PCHC services [23,25,26,28,29,32]. Parents described the postnatal period as a hectic time in which they had to face new insecurities but in which less professional support was provided compared to the pre- and perinatal period [25,26,28,31,32,34]. Parents expressed they did not always feel ready for the steep decrease in provided care and expressed a need for reassurance [25,26,28,29,32]. The sudden change in their situation was a difficult experience to some parents [25,26,32]. To feel secure despite reduced professional support, parents needed to enter the postnatal period well-prepared with easy access to reliable information, reassurance, advice and care [23,25,26,27,29,31,32]. A lack of reliable information on self-care, postnatal care expectations, PCHC services or access to care induced stress and insecurity in parents [23,25,26,28,31,32]. A clear understanding of available services and contact information for providers positively impacted parents’ experience: knowing how to access support servd as a safety net, even if unused [25,26,29,30,31]. Parents experienced difficulties in accessing timely care when needed between scheduled consultations [25,26,29,31,32]. This was partly because of waiting times but also due to the emotional challenge of reaching out for help and not knowing when or where to ask for help [25,26,29,31,32]. Lastly, accessible and reliable information and advice from trained healthcare professionals was reported to help parents feel secure and reassured [23,26,27,29,31,32,35]. Reliable digital information sources as well as a telephone service were mentioned as possible options to provide such a service [23,26,27,29,32].

#### Theme 2: Parents’ wish for monitoring and support of well-being

Both mothers and partners expressed their concerns about insufficient interest in the postnatal well-being of the parents [23,26,27,28,29,32]. They indicated that, after birth, they felt like the focus of health professionals often shifts towards the child, even though childbirth and becoming a parent also greatly impacts the parents themselves [26,27,28,29]. The shift from *maternity* care to *PCHC* aggravates the feeling that the focus shifts away from the parents’ well-being. After discharge of maternity care parents sometimes felt like they could not reach out to the midwife or obstetrician anymore [23,26,27,29]. Since parents may perceive PCHC as solely focused on child care, they may be less aware of its support for their own mental and physical healthcare needs [23,26,27,29]. Parents appreciated when the PCHC professional informed about their well-being [23,26]. Not informing about their well-being negatively impacted their view of PCHC, and their willingness to attend consultations. In stead, a family-oriented care philosophy was preferred [23,26].

The quality of the regular (6-12 weeks) post-partum consultation for the mother also had a great impact if parents felt their physical and emotional needs were met [26,27,28,29,31,32]. Due to inflexible timing, insufficient opportunity to share problems or to discuss the birth with a known midwife or obstetricians, the postpartum consultation did not always met parents’ needs [27,29,30,31]. Specifically, partners reported that they were not offered any follow-up care of mental well-being, even though they felt that they were also impacted by becoming a parent and caring for their child and partner [28,32].

#### Theme 3: Parents’ wish for trusting and relying on care providers

Relational continuity was often highlighted as essential for building trust and experience their careprovider to be well-informed to provide tailored care [23,25,27,29,30,31,32]. Consistently seeing the same professionals was mentioned to foster a sense of predictability, stability and reduce variation in care quality [23,25,31]. It also encouraged seeking support, sharing challenges or negative experiences and feeling confident that professionals would recognize important changes in (emotional) well-being, enabling timely intervention [25,31]. To improve relational continuity suggestions included delaying the withdrawal of midwifery care and ensuring that the regular post-partum consultation is provided by a familiar care provider [23,25,26,28,29,30,32]. Some parents felt ‘left behind’ when midwifery care ended, because they could not rely on a trusted midwife anymore [23,26,27]. Introducing the PCHC professional earlier in pregnancy was another recommendation to establish relational continuity [23,24,27,30,31].

Relational continuity was disrupted not only by the transition from maternity care to PCHC but also within disciplines. Ideally, families should see the same midwife, obstetrician, and PCHC professional during every consultation, even across pregnancies [26,27,29,30,31,32]. To foster trust during the transfer to PCHC, an interested, non-judgmental, and supportive approach was effective [31]. Impersonal or rigid consultations at PCHC could harm trust in both the professional and the healthcare service [23,26]. Some parents viewed PCHC as a ‘frontline social service,’ leading to reluctance in sharing sensitive information [25,31]. The presence of a familiar and trusted maternity care provider during consultations facilitated a positive relationship with the PCHC professional [27,30].

Informational continuity was essential in fostering trust, as adequate information transfer enabled professionals to understand and meet the family’s specific needs, creating a more personalized communication from the start [23,25,30,32]. A lack of knowledge about parents’ histories and preferences could lead to social missteps, damaging trust. Importantly, information transfer only promotes trust after if parents’ consent is obtained respectfully [23,25].

#### Theme 4: Parents’ wish for effective interprofessional collaboration

Collaborative service organization and visible collaboration between healthcare providers were seen as key to reducing a fragmented care experience [24,25,30,31,32,36]. Some parents, however, experienced limited collaboration, often unaware of any communication between providers [24,25,30,31,32,36]. Poorly coordinated care, leading to gaps, duplications, or poorly timed consultations, contributed to experienced fragmentation [25,27,36]. Duplications, in particular, were viewed as “pointless” due to increased home visits or travel to healthcare facilities [25,36]. Parents emphasized the need for effective communication between professionals to ensure coordinated care [24,25,27,30,31,32,36].

A lack of shared information not only affects trust in healthcare providers but also increases parents’ sense of fragmentated care [25,30,31,32,36], as they must often repeat information [25,36]. This repetition can be tiring or anxiety-inducing, especially after a traumatic birth [25,30,31,32,36]. Additionally, parents feared that important information would be missed due to fragmented care [32]. To improve the information transfer and flow between maternity care and PCHC, digital transfer of patient data was suggested as a key solution [25,36]. A stronger connection between services can also help to make sure that parents receive consistent information, as conflicting advice or disagreements among professionals undermined trust [25,29,32,36]. Joint training, streamlined guidelines, and coordinated teamwork were recommended to ensure unified support [24,36]. Parents valued feeling empowered by a cohesive team of caregivers, who communicated well and are all ‘on the same page’ [25] and were positive about combined home visits [24,36]. Jointly providing group care to promote collaboration was suggested, which has the added benefit of creating a peer-network [26,28,36]. Lastly, co-location was suggested as a way to promote teamwork and to refer to each other more easily [24,27] However, the success of co-location relied on active collaboration such as a joint telephone service [24,27].

## Discussion

We aimed to synthesize evidence on what impacts parents’ experience of continuity of care, and how to promote continuity of care within the maternity-PCHC care continuum. Our analysis demonstrated that parents prioritized a sense of security at home, support for their well-being, while having the ability to build trusting relationships with professionals and experiencing effective collaboration between professionals. Parents valued care that is accessible, well-organized, personal, tailored, and family-centred, empowering them to be self-reliant. Suggested practices for improving continuity include reliable information transfer and a thoughtful, non-judgmental approach to building trust despite relational gaps. Furthermore, joint working can boost trust in healthcare providers and support interprofessional collaboration and adequate transfer of information. With the development of integration initiatives in mind, it is promising that a variety of practices seem to contribute to continuity of care, sometimes in multiple ways. For example joint home visits enhance both consistent communication and trust simultaneously [25].

Integrating services is challenging, with several studies indicating that creating a more continuous service experience is difficult to implement [15,34,37,38,39,40,41]. Collaboration across specialisms is complex and requires investment from multiple parties [42]. issues regarding collaboration are persistent; a review of maternity services in England and Wales found that issues with poor coordination identified in 1959, remain today [43]. Poor collaboration might in itself also hinder implementation of healthcare innovations as social and cognitive boundaries between professionals hampered the spread of innovation [44]. Therefore, further research into *how* the imperative to collaborate can be implemented into practice is paramount.

Our review showed that inter-organizational changes can improve experienced continuity of care. Introducing the PCHC professional early during pregnancy might be beneficial as the relationship between patient and caregiver can develop and continue postnatally. However, a challenge for this proposition is that PCHC- and midwifery caregivers work in teams with rotating shifts. Evidence suggests that small maternity care teams enhance the experienced care continuity and perinatal outcomes, but these maternity care models are not yet common practice in Europe [45,46]. Enhancing relational continuity within specialisms could also ease collaboration between them. Exploring group care models like CenteringPregnancy and CenteringParenting may be valuable, as reviews suggest they improve medical outcomes, efficiency, and satisfaction among parents and professionals [47,48,49].

Our review highlights that parents’ support needs were often unmet between maternity care and PCHC consultations [25,26,31,32], reflecting previous literature on the challenges of the postnatal period and the decline in care experienced [34]. Existing services and consultation schedules may not adequately address the evolving needs of new and expanding families [26,27,28,29,31,32,36]. While we have already discussed several ways to help parents feel more secure in between consultations, it is valuable to further explore this perceived gap in care. This is particularly important, as a review on interprofessional collaboration found unclear divisions of responsibility during the postnatal period [15].

Specifically regarding the support of parents’ well-being there is an important gap to address. Our review found that professional support often shifts focus from the mother and unborn child to the child after birth, raising concerns about ongoing maternal well-being [26,27,28,29,31,32,36]. Current postnatal care for mothers is infrequent and delayed compared to the antenatal period [34]. In our review we suggested that both maternity care and PCHC services can contribute to improving parental support, which is reflected by previous literature which stated that a holistic cooperation with the family and personalized care at PCHC services is very important to help parents feel supported and can to a high degree determine how the parents view PCHC [34,50,51]. Furthermore, PCHC can also aid in screening for postpartum depression (PPD), as evidence supports its effectiveness [52,53] Repeated assessments are crucial, given the impact of PPD on a child’s socioemotional development [54]. This review also underpins the results of studies highlighting the importance of continuous and accessible availability of maternity care up to 28 days post-partum to support mothers’ well-being [55,56].

## Strengths and limitations

An important strength of this review is the multidisciplinary research team, including researchers with a background in maternity care, PCHC, organization and management and sociology, ensuring a comprehensive perspective. The qualitative nature of the included studies captured diverse parental experiences and the values shaping them. However, we could not fully account for country-specific contexts, which could have strengthened the study. This review mainly synthesized common themes to highlight key issues for integration efforts, limiting our ability to delve into the specific complexities discussed in individual studies.

## Conclusions

Ideally, (a) parents enter the postnatal period well-prepared, and well-informed about self-care, PCHC and possible postnatal carepathways, (b) number of caretransfers is limited, (c) by overlapping maternity care and PCHC, parents are provided with an opportunity to maintain meaningful relationships with their care providers, and (d) information is consistent, family-centred, and tailored.

## Additional File

The additional file for this article can be found as follows:

10.5334/ijic.8645.s1 Appendix A. Search strategy and SRQR quality assessment.
