# Supplementary material for: Parent’s Perspective on Continuity of Care in the Maternity Care and Child Health Services Continuum: A Qualitative Systematic Review
Source: Int J Integr Care. 2025 Jan 24;25(1):4. doi: 10.5334/ijic.8645 (PMC11758814; doi:10.5334/ijic.8645)
Supplement: Appendix A. — Search strategy and SRQR quality assessment. [file ijic-25-1-8645-s1.pdf]

Appendix A

Search strategy

Research question

'What is important to new mothers with regard to continuity across the maternity and child and family health service continuum?'

Strategy

Database searched	via	Years of coverage	References	After de-duplication
Embase	Embase.com	1971 - Present	4923	4869
Medline ALL	Ovid	1946 - Present	3001	976
Web of Science Core Collection*	Web of Knowledge	1975 - Present	1565	275
Cochrane Central Register of Controlled Trials**	Wiley	1992 - Present	522	242
CINAHL	EBSCO	1982 - Present	1634	438
Other sources: Google Scholar			200	193
**Total**			**11845**	**6993**

*Science Citation Index Expanded (1975-present) ; Social Sciences Citation Index (1975-present) ; Arts & Humanities Citation Index (1975-present) ; Conference Proceedings Citation Index- Science (1990-present) ; Conference Proceedings Citation Index- Social Science & Humanities (1990-present) ; Emerging Sources Citation Index (2005-present)

** Manually deleted abstracts from trial registries

All terms added in May 2022 are marked yellow.

Embase.com

('patient care'/de OR 'clinical handover'/de OR (('health service'/exp OR 'health center'/de OR 'maternal health service'/de) AND ('cooperation'/de)) OR 'maternal child health care'/de OR (handoff* OR hand-off* OR handover OR sign-out* OR sign-over* OR signover* OR sign-off* OR signoff OR signout* OR intershift* OR ((chang*) NEAR/3 (shift*)) OR ((collaborat* OR relation* OR co-operat* OR cooperat* OR communicat* OR continu*) NEAR/3 (health* OR care* OR network* OR transit* OR transfer*)) OR ((integrat* OR interprofession* OR inter-profession* OR interdisciplin* OR inter-disciplin* OR multidisciplin* OR multi-disciplin* OR crossdisciplin* OR cross-disciplin* OR transdisciplin* OR trans-disciplin* OR intersector* OR inter-sector*) NEAR/3 (collaborat* OR co-operat* OR cooperat* OR communicat* OR healthcar* OR care*)) OR IMPAC OR MNCH OR MCH OR MCHC OR MHC OR ((mother*) NEAR/3 (child*) NEAR/3 (health* OR care*)) OR center*-pregnan* OR (group* NEAR/3 care*) OR health-visit* OR preventive-child-health-care* OR child-health-care-services* OR matern*-service* OR early-support* OR ((interpersonal* OR inter-personal* OR geographical* OR chronological* OR longitudinal* OR relational* OR management* OR information* OR organization* OR organisation* OR team* OR cross-boundar* OR experience* OR seamless* OR integrat* OR coordinat* OR co-ordinat*) NEAR/3 (continu*)) OR ((coordinat* OR co-ordinat*) NEAR/3 (deliver*)) OR ((child* OR famil* OR well-baby OR maternal* OR communit* OR public*)

NEAR/3 (clinic* OR health-service* OR health-nurse*))):ab,ti,kw) **AND** ('newborn'/de OR 'postnatal care'/exp OR (newborn* OR new-born* OR post-natal* OR postnatal* OR post-birth* OR after-birth* OR postpartum* OR after-pregnan* OR well-baby OR well-babies OR neonate OR neonates):ab,ti,kw) **AND** ('satisfaction'/de OR 'patient satisfaction'/de OR 'health care quality'/de OR 'patient attitude'/exp OR 'attitude to health'/de OR (((patient* OR family OR families OR parent* OR mother* OR service-user* OR woman OR women OR father* OR partner*) NEAR/3 (value* OR experience* OR perspective* OR attitude* OR outlook* OR position* OR view* OR voice* OR importan* OR satisf* OR benefi*))):ab,ti,kw) *NOT [conference abstract]/lim*

Medline

(Continuity of Patient Care/ OR Patient Handoff/ OR ((Health Services/ OR Maternal Health Services/) AND (Intersectoral Collaboration/ OR Cooperative Behavior/)) OR Maternal-Child Health Centers/ OR (handoff* OR hand-off* OR handover OR sign-out* OR sing-over* OR singover* OR sing-off* OR singoff OR signout* OR intershift* OR ((chang*) ADJ3 (shift*)) OR ((collaborat* OR relation* OR co-operat* OR cooperat* OR communicat* OR continu*) ADJ3 (health* OR care* OR network* OR transit* OR transfer*)) OR ((integrat* OR interprofession* OR inter-profession* OR interdisciplin* OR inter-disciplin* OR multidisciplin* OR multi-disciplin* OR crossdisciplin* OR cross-disciplin* OR transdisciplin* OR trans-disciplin* OR intersector* OR inter-sector*) ADJ3 (collaborat* OR co-operat* OR cooperat* OR communicat* OR healthcar* OR care*)) OR IMPAC OR MNCH OR MCH OR MCHC OR MHC OR ((mother*) ADJ3 (child*) ADJ3 (health* OR care*)) OR center*-pregnan* OR (group* ADJ3 care*) OR health-visit* OR preventive-child-health-care* OR child-health-care-services* OR matern*-service* OR early-support* OR ((interpersonal* OR inter-personal* OR geographical* OR chronological* OR longitudinal* OR relational* OR management* OR information* OR organization* OR organisation* OR team* OR cross-boundar* OR experience* OR seamless* OR integrat* OR coordinat* OR co-ordinat*) ADJ3 (continu*)) OR ((coordinat* OR co-ordinat*) ADJ3 (deliver*)) OR ((child* OR famil* OR well-baby OR maternal* OR communit* OR public*) ADJ3 (clinic* OR health-service* OR health-nurse*))).ab,ti,kf.) **AND** (Infant, Newborn/ OR Postnatal Care/ OR (newborn* OR new-born* OR post-natal* OR postnatal* OR post-birth* OR after-birth* OR postpartum* OR after-pregnan* OR well-baby OR well-babies OR neonate OR neonates).ab,ti,kf.) **AND** (Personal Satisfaction/ OR Patient Satisfaction/ OR Quality of Health Care/ OR Attitude to Health/ OR (((patient* OR family OR families OR parent* OR mother* OR service-user* OR woman OR women OR father* OR partner*) ADJ3 (value* OR experience* OR perspective* OR attitude* OR outlook* OR position* OR view* OR voice* OR importan* OR satisf* OR benefi*))).ab,ti,kf.) *NOT (news OR congres* OR abstract* OR book* OR chapter* OR dissertation abstract*).pt.*

Cochrane Central

((handoff* OR hand NEXT off* OR handover OR sign NEXT out* OR sign NEXT over* OR signover* OR sign NEXT off* OR signoff OR signout* OR intershift* OR ((chang*) NEAR/3 (shift*)) OR ((collaborat* OR relation* OR co NEXT operat* OR cooperat* OR communicat* OR continu*) NEAR/3 (health* OR care* OR network* OR transit* OR transfer*)) OR ((integrat* OR interprofession* OR inter NEXT profession* OR interdisciplin* OR inter NEXT disciplin* OR multidisciplin* OR multi NEXT disciplin* OR crossdisciplin* OR cross NEXT disciplin* OR transdisciplin* OR trans NEXT disciplin* OR intersector* OR inter NEXT sector*) NEAR/3 (collaborat* OR co NEXT operat* OR cooperat* OR

communicat* OR healthcar* OR care*)) OR IMPAC OR MNCH OR MCH OR MCHC OR MHC OR ((mother*) NEAR/3 (child*) NEAR/3 (health* OR care*)) OR center* NEXT pregnan* OR (group* NEAR/3 care*) OR health NEXT visit* OR preventive NEXT child NEXT health NEXT care* OR child NEXT health NEXT care NEXT services* OR matern* NEXT service* OR early NEXT support* OR ((interpersonal* OR inter NEXT personal* OR geographical* OR chronological* OR longitudinal* OR relational* OR management* OR information* OR organization* OR organisation* OR team* OR cross NEXT boundar* OR experience* OR seamless* OR integrat* OR coordinat* OR co NEXT ordinat*) NEAR/3 (continu*)) OR ((coordinat* OR co NEXT ordinat*) NEAR/3 (deliver*)) OR ((child* OR famil* OR well NEXT baby OR maternal* OR communit* OR public*) NEAR/3 (clinic* OR health NEXT service* OR health NEXT nurse*))):ab,ti,kw) **AND** ((newborn* OR new NEXT born* OR post NEXT natal* OR postnatal* OR post NEXT birth* OR after NEXT birth* OR postpartum* OR after NEXT pregnan* OR well NEXT baby OR well NEXT babies OR neonate OR neonates):ab,ti,kw) **AND** ((((patient* OR family OR families OR parent* OR mother* OR service NEXT user* OR woman OR women OR father* OR partner*) NEAR/3 (value* OR experience* OR perspective* OR attitude* OR outlook* OR position* OR view* OR voice* OR importan* OR satisf* OR benefi*))):ab,ti,kw) NOT "Conference Abstract":pt

Web of Science

TS=(((handoff* OR hand-off* OR handover OR sign-out* OR sign-over* OR signover* OR sign-off* OR signoff OR signout* OR intershift* OR ((chang*) NEAR/2 (shift*)) OR ((collaborat* OR relation* OR co-operat* OR cooperat* OR communicat* OR continu*) NEAR/2 (health* OR care* OR network* OR transit* OR transfer*)) OR ((integrat* OR interprofession* OR inter-profession* OR interdisciplin* OR inter-disciplin* OR multidisciplin* OR multi-disciplin* OR crossdisciplin* OR cross-disciplin* OR transdisciplin* OR trans-disciplin* OR intersector* OR inter-sector*) NEAR/2 (collaborat* OR co-operat* OR cooperat* OR communicat* OR healthcar* OR care*)) OR IMPAC OR MNCH OR MCH OR MCHC OR MHC OR ((mother*) NEAR/2 (child*) NEAR/2 (health* OR care*)) OR center*-pregnan* OR (group* NEAR/2 care*) OR health-visit* OR preventive-child-health-care* OR child-health-care-services* OR matern*-service* OR early-support* OR ((interpersonal* OR inter-personal* OR geographical* OR chronological* OR longitudinal* OR relational* OR management* OR information* OR organization* OR organisation* OR team* OR cross-boundar* OR experience* OR seamless* OR integrat* OR coordinat* OR co-ordinat*) NEAR/2 (continu*)) OR ((coordinat* OR co-ordinat*) NEAR/2 (deliver*)) OR ((child* OR famil* OR well-baby OR maternal* OR communit* OR public*) NEAR/2 (clinic* OR health-service* OR health-nurse*)))) **AND** ((newborn* OR new-born* OR post-natal* OR postnatal* OR post-birth* OR after-birth* OR postpartum* OR after-pregnan* OR well-baby OR well-babies OR neonate OR neonates)) **AND** ((((patient* OR family OR families OR parent* OR mother* OR service-user* OR woman OR women OR father* OR partner*) NEAR/2 (value* OR experience* OR perspective* OR attitude* OR outlook* OR position* OR view* OR voice* OR importan* OR satisf* OR benefi*))))) AND DT=(Article OR Review OR Early Access OR Letter)

CINAHL

(MH “Continuity of Patient Care” OR MH “Hand Off (Patient Safety)” OR ((MH “Health Services” OR MH “Maternal Health Services”) AND (MH Collaboration OR MH “Cooperative Behavior”)) OR MH “Maternal-Child Health” OR TI (handoff* OR hand-off* OR handover OR sign-out* OR sing-over* OR singover* OR sing-off* OR singoff OR signout* OR intershift* OR ((chang*) N2 (shift*)) OR

((collaborat* OR relation* OR co-operat* OR cooperat* OR communicat* OR continu*) N2 (health* OR care* OR network* OR transit* OR transfer*)) OR ((integrat* OR interprofession* OR inter-profession* OR interdisciplin* OR inter-disciplin* OR multidisciplin* OR multi-disciplin* OR crossdisciplin* OR cross-disciplin* OR transdisciplin* OR trans-disciplin* OR intersector* OR inter-sector*) N2 (collaborat* OR co-operat* OR cooperat* OR communicat* OR healthcar* OR care*)) OR IMPAC OR MNCH OR MCH OR MCHC OR MHC OR ((mother*) N2 (child*) N2 (health* OR care*)) OR center*-pregnan* OR (group* N2 care*) OR health-visit* OR preventive-child-health-care* OR child-health-care-services* OR matern*-service* OR early-support* OR ((interpersonal* OR inter-personal* OR geographical* OR chronological* OR longitudinal* OR relational* OR management* OR information* OR organization* OR organisation* OR team* OR cross-boundar* OR experience* OR seamless* OR integrat* OR coordinat* OR co-ordinat*) N2 (continu*)) OR ((coordinat* OR co-ordinat*) N2 (deliver*)) OR ((child* OR famil* OR well-baby OR maternal* OR communit* OR public*) N2 (clinic* OR health-service* OR health-nurse*))) OR AB (handoff* OR hand-off* OR handover OR sign-out* OR sing-over* OR singover* OR sing-off* OR singoff OR signout* OR intershift* OR ((chang*) N2 (shift*)) OR ((collaborat* OR relation* OR co-operat* OR cooperat* OR communicat* OR continu*) N2 (health* OR care* OR network* OR transit* OR transfer*)) OR ((integrat* OR interprofession* OR inter-profession* OR interdisciplin* OR inter-disciplin* OR multidisciplin* OR multi-disciplin* OR crossdisciplin* OR cross-disciplin* OR transdisciplin* OR trans-disciplin* OR intersector* OR inter-sector*) N2 (collaborat* OR co-operat* OR cooperat* OR communicat* OR healthcar* OR care*)) OR IMPAC OR MNCH OR MCH OR MCHC OR MHC OR ((mother*) N2 (child*) N2 (health* OR care*)) OR center*-pregnan* OR (group* N2 care*) OR health-visit* OR preventive-child-health-care* OR child-health-care-services* OR matern*-service* OR early-support* OR ((interpersonal* OR inter-personal* OR geographical* OR chronological* OR longitudinal* OR relational* OR management* OR information* OR organization* OR organisation* OR team* OR cross-boundar* OR experience* OR seamless* OR integrat* OR coordinat* OR co-ordinat*) N2 (continu*)) OR ((coordinat* OR co-ordinat*) N2 (deliver*)) OR ((child* OR famil* OR well-baby OR maternal* OR communit* OR public*) N2 (clinic* OR health-service* OR health-nurse*)))) **AND** (MH "Infant, Newborn"+ OR MH "Postnatal Care" OR TI (newborn* OR new-born* OR post-natal* OR postnatal* OR post-birth* OR after-birth* OR postpartum* OR after-pregnan* OR well-baby OR well-babies OR neonate OR neonates) OR AB (newborn* OR new-born* OR post-natal* OR postnatal* OR post-birth* OR after-birth* OR postpartum* OR after-pregnan* OR well-baby OR well-babies OR neonate OR neonates)) **AND** (MH "Personal Satisfaction" OR MH "Patient Satisfaction" OR MH "Quality of Health Care" OR MH "Attitude to Health" OR TI (((patient* OR family OR families OR parent* OR mother* OR service-user* OR woman OR women OR father* OR partner*) N2 (value* OR experience* OR perspective* OR attitude* OR outlook* OR position* OR view* OR voice* OR importan* OR satisf* OR benefi*))) OR AB (((patient* OR family OR families OR parent* OR mother* OR service-user* OR woman OR women OR father* OR partner*) N2 (value* OR experience* OR perspective* OR attitude* OR outlook* OR position* OR view* OR voice* OR importan* OR satisf* OR benefi*))))

Google Scholar

'continuity|continuation healthcare|care'|'mother child health|care'|IMPAC|MNCH|MCH|MCHC newborn|'after birth'|well-baby|well-babies|neonate|neonates

'patient|family|families|parent|mother|father value|experience|perspective|attitude|satisfaction'

Supplemental table 1 – Quality assessment according to the Standards for Reporting Qualitative Research (SRQR) checklist.

Topic	Barimani and Hylander (2012)	Barimani et al. (2015)	Vikström and Barimani (2016)	Woordward, Zadoroznj, Benoit (2016)	Franck, McNulty and Alderdice (2017)	Aquino, Olander and Bryar (2018)	Olander et al. (2019)	Olander, Aquino and Bryar (2019)	Forss, Mangrio and Hellström (2022)	Frederiksen, Schmied, Overgaard (2022)
**Title**						X	X	X		X
**Abstract**	X	X	X	X	X	X	X	X	X	X
**Problem formulation**	X	X	X	X	X	X	X	X	X	X
**Purpose or research question**	X	X	X	X	X	X	X	X	X	X
**Qualitative approach and research paradigm**	X	X				X				X
**Researcher characteristics, reflexivity**		X	X			X		X		X
**Context**	X	X	X	X	X	X	X	X	X	X
**Sampling strategy**	X		X	X	X	X		X	X	X
**Ethical issues pertaining to human subjects**	X	X	X	X	X	X	X	X	X	X
**Data collection methods**	X	X	X	X	X	X	X	X	X	X
**Data collection instruments/ technologies**	X	X	X	X	X	X	X	X	X	X
**Units of study**	X	X	X	X	X	X	X	X	X	X
**Data processing**	X	X	X	X	X	X	X	X		

**Data analysis**	X	X	X	X	X	X	X	X	X	X
**Techniques to enhance trustworthiness**	X	X		X	X	X	X	X	X	X
**Synthesis and interpretation**	X	X	X	X	X	X	X	X	X	X
**Links to empirical data**	X	X	X	X	X	X	X	X	X	X
**Integration with prior work, implications, transferability, and contributions**	X	X	X	X	X	X	X	X	X	X
**Limitations**	X	X	X	X	X	X	X	X	X	X
**Conflicts of interest**	X	X	X		X	X	X	X		X
**Funding**	X	X	X			X	X	X	X	X
